# Detection of Amyloid-β42 Using a Waveguide-Coupled Bimetallic Surface Plasmon Resonance Sensor Chip in the Intensity Measurement Mode

**DOI:** 10.1371/journal.pone.0098992

**Published:** 2014-06-09

**Authors:** Yeon Kyung Lee, Kyeong-Seok Lee, Won Mok Kim, Young-Soo Sohn

**Affiliations:** 1 Department of Biomedical Engineering, Catholic University of Daegu, Gyeongsan-si, Gyeongbuk, Republic of Korea; 2 Electronic Material Research Center, Korea Institute of Science and Technology, Seoul, Republic of Korea; University of California, Merced, United States of America

## Abstract

The waveguide-coupled bimetallic (WcBiM) surface plasmon resonance (SPR) chip had been utilized in the intensity interrogation detection mode to detect amyloid-β42 (Aβ42), a biomarker of the Alzheimer disease. The SPR reflectance curve of the WcBiM chip has the narrower full-width-at-half-maximum (FWHM) compared with the SPR reflectance curve of the conventional gold (Au) chip, resulting in the steeper gradient. For the enhancement of resolution, the light source was fixed at an angle where the slope of the reflectance curve is the steepest, and the change in the reflectance was monitored. For the detection of Aβ42, the antibody of Aβ42 (anti-Aβ42) was immobilized on the WcBiM SPR chip using the self-assembled monolayer. The SPR responses, the average changes in the reflectance to the Aβ42 at the concentrations of 100 pg/ml, 250 pg/ml, 500 pg/ml, 750 pg/ml, 1,000 pg/ml, and 2,000 pg/ml were 0.0111%, 0.0305%, 0.0867%, 0.1712%, 0.3021%, and 0.5577%, respectively, for the three replicates. From linear regression analysis, the calibration curve indicated that the SPR response had a linear relation with Aβ42 with the concentration in the range of 100 pg/ml to 2,000 pg/ml. A control experiment showed the anti-Aβ42-modified surface of the WcBiM chip had a high specificity to Aβ42. Thus, the enhanced resolution by utilizing the WcBiM SPR chip in the intensity interrogation detection mode aids the diagnosis of the Alzheimer disease by detecting the Aβ42 around the criteria concentration (500 pg/ml) without any labeling.

## Introduction

The rapid development of medical technologies has led to an aging society, and the rate of the incidence of dementia is increasing [1]. Throughout the world, Alzheimer disease (AD) is one of the most frequently diagnosed types of dementias. It is estimated that about 80 million people across the globe will be diagnosed with AD by 2,050 [2], [3], and the AD-related social medical costs will increase. Recently, the clinical paradigm is being transferred from treatment after occurrence to disease prevention or early treatment. Thus, early diagnosis of diseases, including AD, is emphasized for successful prognosis and prevention. Early identification and monitoring of diseases are essential for maintaining a high quality of human life. A high quality of life can be also attributed to better treatment, improvements in survivability, and low medical costs [4], [5]. Biosensors provide fast and accurate detection and can serve as a crucial tool in the early diagnosis of a disease [6]–[8].

The prognosis for AD is dependent upon the identification of intracellular neurofibrillary tangles (NFTs) and extracellular senile plaques, resulting in neuronal dysfunction and cell death. NFTs are insoluble fibers found in the brain's nerve cells. These NFTs are the result of abnormal hyperphosphorylation of the microtubule-associated protein tau (τ) and they are deposited inside of the neurons, resulting in the disruption of the neurons [9]. One of the major constituents of the senile plaques is amyloid-β (Aβ) which is proteolytically cleaved from amyloid precursor protein (APP). The major Aβ species generated from APP are 40-amino-acid (Aβ40) or 42-amino-acid (Aβ42) peptides, inducing neuronal death [3], [9], [10]. In addition, Aβ40 and Aβ42 have the property of toxicity due to the presence of a single methionine 35 in their amino acid sequences. Moreover, methionine 35 is highly prone to oxidation under conditions of oxidative stress which, in turn, causes cell death in neurons [11], [12]. Especially, Aβ42 has two additional residues at the carboxyl-terminal of Aβ40 and a propensity towards high self-aggregation into the plagues. Thus, Aβ42 is more vulnerable to aggregation than Aβ40 [9]. Furthermore, Aβ42 produces neuritic plaques inside the cells of the brain and leads to the damage of the neuronal processes and synapses for approximately 10–15 years before the pathogenesis of AD. Increases in the amount of Aβ42 plague inside the brain's cells or decreases in the amount of Aβ42 in cerebrospinal fluid (CSF) indicate the onset of AD [13]. It is reported that the AD patients have the level of Aβ42 less than 500 pg/ml in CSF [3], [14]. Therefore, Aβ42 is a very useful biomarker for confirming whether the patient has a good or bad prognosis for AD. Since the quality of life is extremely terrible after the pathogenesis of AD, early detection of Aβ42 is very important for treating this disease.

Measurements of Aβ42 or the other AD-related targets for AD are traditionally performed using enzyme linked immunosorbent assay (ELISA) [15], [16], mass spectrometry (MS) assay [17] and scanning tunneling microscopy (STM) [18]. These techniques require the double antibody technique and the chemical process of query molecules with labels. The labelling process imposes additional time and demand for cost, and can interfere during the biomolecular interaction by blocking a binding site [19], [20]. For the detection of the AD-related biomarker, the surface plasmon resonance (SPR) sensor has been extensively researched since it does not require the labeling process and double antibody technique, and can detect natural forms of query molecules [21], [22]. The SPR biosensor is an extremely sensitive tool that can be used to analyze biomolecular interactions in real-time, responding to minute variations in the refractive index due to the adsorption of the trace level concentration of biomolecules on the metal surface [23]–[26]. The SPR biosensor has the advantages of ultra-high sensitivity, label-free detection, and real-time monitoring. The label-free method can preserve original characteristic of analyte and is effective to reduce time or cost since it does not require any additional chemical process. And it is relatively simple to monitor the biomolecular interactions. In addition, though the SPR sensor is based on optics, it works in turbid or opaque samples. Surface plasmon (SP) is a collective electron oscillation that occurs at the interface between any two materials, primarily a dielectric medium and a metallic medium, which is stimulated by an incident light. SPR occurs when the incident wave vector is matched with the SP wave vector at a certain incident angle where the reflected light significantly decreases, giving rise to a sharp dip in the SPR reflectance curve [27]. This specific angle is referred to as a resonance angle, which has a reflectance minimum. In the Kretschmann configuration the resonance angle is dependent upon the refractive index of the sample medium since the refractive index of the prism does not change. The metals considered for the SPR phenomenon are gold (Au), sliver (Ag), aluminum (Al), and copper (Cu) [28]. In general, Au and Ag are the two main metals used for SPR sensor applications in the visible wavelength region. Due to its chemical stability and biocompatibility, Au is widely adopted for use as the SPR sensor chip. Ag is known for having a narrower spectral width in the SPR reflectance curve [29]. However, Ag has a poor chemical stability because it is highly vulnerable to oxidation when it is exposed to air or liquid environments [28]. Since the detection of biomolecules with a trace level concentration or a very low molecular weight is challenging, a SPR sensor based on the bimetallic Ag/Au films (Au as an outer layer) was proposed in order to take advantage of both films, thereby providing the higher signal-to-noise ratio of the system [29]–[32]. In an Ag-Au bimetallic chip, the full width at half maximum (FWHM) is narrower than it is in a conventional Au chip, resulting in a more change in the reflectance in the intensity interrogation detection mode. In addition, the bimetallic film coupled with a waveguide layer has also been proposed, since the incident light field is coupled into the inner metal layer interfacing with the prism and then propagated to the outer metal layer through the waveguide layer, with minimizing optical field leakage into the sensing region [33], [34]. The line width of the reflectance curve of the waveguide-coupled bimetallic (WcBiM) film is narrower than the line widths of the conventional Au and bimetallic films, thus enhancing the sensitivity in the intensity interrogation detection scheme [35], [36].

In this paper, the WcBiM SPR chip has been proposed to detect Aβ42 for the diagnosis of AD. The configuration of the WcBiM SPR chip has a high-index dielectric waveguide sandwiched between two metal layers: Ag as the inner metal layer and Au as the outer metal layer. After acquiring the reflectance curve of the WcBiM SPR chip, the working point was set to the angle of the incident light with the steepest slope of the SPR reflectance curve to maximize the resolution in the intensity detection mode. For the detection of Aβ42, antibody of Aβ42 (anti-Aβ42) was covalently immobilized on the WcBiM SPR chip surface through a self-assembled monolayer (SAM). The WcBiM SPR response to various concentrations of Aβ42 was presented, and a calibration curve was acquired. A control experiment was also carried out to investigate the specificity of the anti-Aβ42-modified surface of the WcBiM chip.

## Methods and Materials

### Surface plasmon resonance sensor

**Figure 1:**
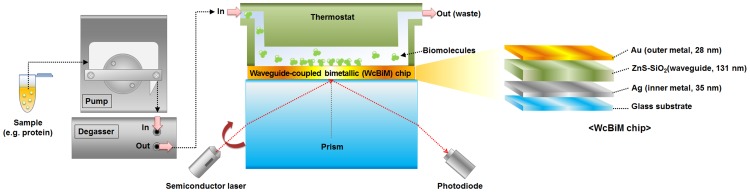
Schematic diagram of the SPR sensor system and the WcBiM chip configuration.

A schematic of the SPR sensor system (SPR LAB, K-MAC; Daejeon, Korea) based on the Kretschmann configuration is presented in Figure 1. As can be seen, the incident p-polarized light of a semiconductor laser (635 nm, 2.5 mW) is directed onto the WcBiM chip through a rectangular parallelepiped prism (18 mm (W) ×18 mm (D) ×10 mm (H)), and the reflected light is detected by a photodiode detector. The sensor system includes a thermostat to exclude the effect of ambient temperature change. All the experiments were carried out at 25°C. The SPR reflectance curve was acquired by scanning the laser with the incident angle range of 30°–80° via a pulse motor. The optimized incident angle at which the gradient was the steepest in the SPR reflectance curve was found by differentiating the reflectance curve with respect to the incident angle for the intensity interrogation detection scheme. The light source was fixed at this angle, and then the reflectance was monitored. All the proteins were injected into the fluidic module of the SPR sensor system after removing the air bubble by using a degasser. A constant flow rate of 7.5 µl/min was maintained throughout the duration of all of the experiments.

### Detection Principle

**Figure 2:**
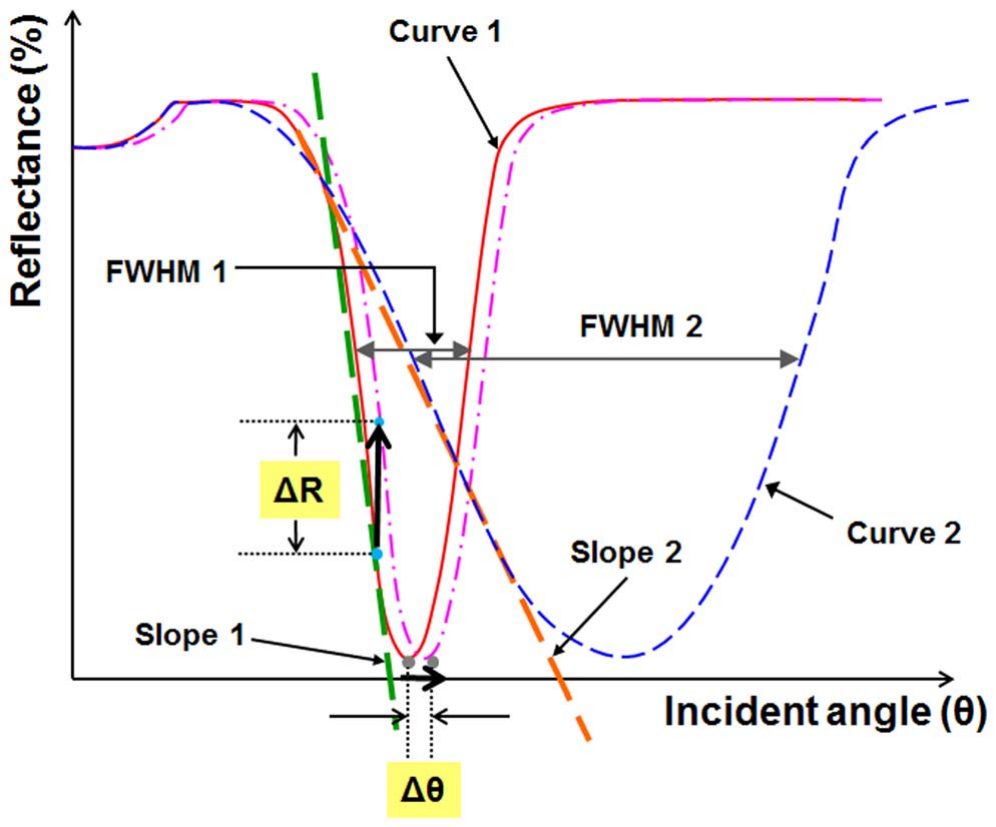
Both narrow and wide reflectance curves and their corresponding slopes and FWHMs.

Four different SPR detection schemes—angular interrogation, intensity interrogation, wavelength interrogation, and phase interrogation—are used in SPR sensor applications. Due to the limited material choice of the metal film much effort has been made to decrease the FWHM of the reflectance curve in the intensity interrogation detection scheme for improvement in the sensor resolution [32], [34], [35]-[37]. The detection principle of the intensity measurement scheme is that the light source is fixed at the angle where the slope is the steepest in the reflectance curve, and then the reflectance is measured. In general, the steeper the slope of the reflectance curve, the bigger the difference in the reflectance at the same refractive index change. Thus, in order to improve the resolution in the intensity measurement scheme, one can decrease the FWHM of the reflectance curve to obtain the steeper slope, as shown in Figure 2. As the FWHM of the reflectance curve decreases, the absolute value of the gradient in the reflectance curve increases. The angle where the slope is the steepest can be found by differentiating the reflectance curve with respect to the incident angle. Then, the light source is fixed at this angle, and the reflectance is monitored. When biomolecular interactions occur on the SPR chip surface, the reflectance curve, in general, shifts to the right, as shown in Figure 2 (from a solid line to a dot-and-dash line). At this fixed angle mode, a fairly handsome margin of the reflectance can be acquired on the biomolecular interactions, and the resolution of the intensity measurement mode is better than it is on the angular interrogation detection mode [36].

To decrease the FWHM of the reflectance curve, the bimetallic films composed of Au and Ag, two main materials for the SPR sensor applications, are adopted [29], [31], [32], [38], [39]. As the Ag/Au ratio of the bimetallic film increases, the FWHM of the reflectance curve decreases resulting in an increase in the sensitivity. A distinct configuration in which a waveguide is coupled with the bimetallic film was proposed [34]–[36], and the FWHM of the reflectance curve for this WcBiM film was narrower than it was for the bimetallic films composed of Au and Ag as shown in Figure S1. Thus, WcBiM SPR chips are expected to have a higher sensitivity than both conventional Au and bimetallic SPR chips [36].

### Waveguide-coupled bimetallic (WcBiM) chip

As shown in Figure 1, the WcBiM sensor chip consists of a glass substrate (Corning glass with refractive index n_p_ = 1.508), an Ag inner-metal layer (n_Ag_ = 0.0557+*i*4.2931 at 635 nm), a dielectric waveguide (ZnS-SiO_2_, n_d_ = 2.16784+*i*0.00204 at 635 nm), and an Au outer-metal layer (n_Au_ = 0.18016+*i*3.4531 at 635 nm). The introduction of the Ag inner layer creates a sharper SPR reflectance curve because Ag metal yields better enhancement of the evanescent field. The Au outer surface retains its chemical stability and biocompatibility properties. As a waveguide, ZnS-SiO_2_ was adopted between the Ag and Au layers due to its ability to provide good adhesion between these layers. The thickness of the WcBiM chip was optimized using commercial optical thin film software (SCI Film Wizard). The optimized thicknesses of the Ag, ZnS-SiO_2_, and Au layers were 35 nm, 131 nm, and 28 nm, respectively. The WcBiM chip was fabricated on the Corning glass substrate (12 mm (W) ×12 mm (D) ×0.3 mm (H)) by RF magnetron sputtering with 5 mTorr of working pressure in an Ar atmosphere. The RF power for the ZnS-SiO_2_ and for both the Au and Ag layers was set to 80 W and 20 W, respectively.

### Materials

Aβ42 and anti-Aβ42 were purchased from Millipore (Billerica, MA, USA). Phosphate buffered saline (PBS), bovine serum albumin (BSA) and Aβ40 were obtained from Sigma-Aldrich (St. Louis, MO, USA). The PBS solution was used to dilute all the proteins. In addition, the SAM, used as a linkage layer to immobilize the anti-Aβ42, was purchased from K-MAC (Daejeon, Korea).

### Detection of Aβ42

For the immobilization of anti-Aβ42, the surface modification of the WcBiM chip was carried out by utilizing the SAM. The SAM contains the following crosslinking materials: 1-ethyl–3-(dimethylaminopropyl) carbodiimide hydrochloride (EDC) and N-Hydroxysuccinimide (NHS). EDC and NHS were used for a covalent binding strategy, which forms an amide bond between a carboxyl group and an amine group. Thus, for the immobilization of anti-Aβ42, the SAM was formed onto the WcBiM chip surface by immersing it into a solution of 1 mM SAM in chloroform for 12 hours. Then, the anti-Aβ42 solution was flowed onto the WcBiM chip to immobilize anti-Aβ42. Next, 100 µg/ml of BSA was injected onto the WcBiM chip to prevent a non-specific reaction. Finally, Aβ42, at concentrations of 100 pg/ml, 250 pg/ml, 500 pg/ml, 750 pg/ml, 1,000 pg/ml, and 2,000 pg/ml was separately injected into the WcBiM chip. To prove selectivity, a control test was carried out by using Aβ40 instead of Aβ42. The flow rate was 7.5 µl/min, and the volume of all the proteins was 150 µl.

## Results and Discussion

### High-density immobilization of anti-Aβ42

The high-density immobilization of the biorecognition element on the sensor chip is critical in order to enhance the sensitivity of the sensor since doing so provides a higher probability that the binding events between the analyte and the biorecognition element will occur. Since the outer surface of the WcBiM is comprised of Au metal, which is exactly the same metal that is used for the commercialized Au chip, the commercialized Au chip was utilized to carry out the high-density immobilization test of anti-Aβ42 with another SPR sensor (SPRmicro, K-MAC; Daejeon, Korea) in order to help insure easy data acquisition. The anti-Aβ42 solutions, with concentrations ranging from 10 µg/ml to 200 µg/ml, were flowed over the SAM-formed sensor chip at a flow rate of 30 µl/min. The responses of the SPR sensor to the concentration of anti-Aβ42 ranging from 10 µg/ml to 200 µg/ml are shown in Figure S2. From the curve fitting, 150 µg/ml of the anti-Aβ42 enables the high-density immobilization considering the saturated value. In our experiments, we utilized 150 µg/ml of concentration for the immobilization of the anti-Aβ42 with a much slower injection flow rate.

### SPR reflectance curve

**Figure 3:**
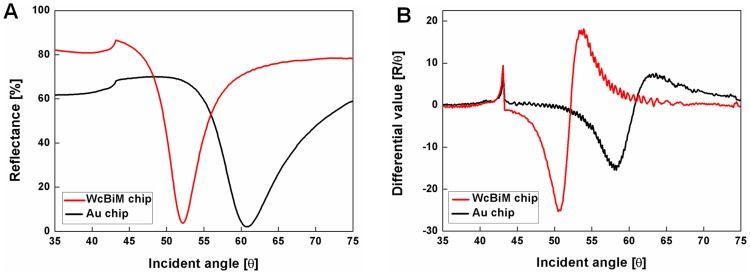
SPR reflectance curves and their derivatives of WcBiM and Au chips. A) SPR reflectance curve for the WcBiM chip in comparison to the conventional Au chip. B) Derivatives of the reflectance curves with respect to the incident angle for both the WcBiM chip and the Au chip.

The SPR reflectance curve for the WcBiM chip was acquired in order to compare it with the SPR reflectance curve for the conventional Au chip. The SPR reflectance curves for the WcBiM chip and the conventional Au chip are presented in Figure 3A. The minimum reflectance at the resonance angle for both the WcBiM chip and the Au chip was 3.7095% at 52.2° and 2.0549% at 60.8°, respectively. As shown in Fig. 3a, the line width of the WcBiM reflectance curve is narrower than the line width of the Au reflectance curve. The FWHMs in the SPR reflectance curves for both the WcBiM and Au chips were 5.2° and 9.5°, respectively. The FWHM of the Au chip was about 1.8 times wider than the FWHM of the WcBiM chip. Figure 3B shows the derivative of the reflectance curve with respect to the incident angle. The maximum slopes (ΔR/Δθ [%/°]) in the SPR reflectance curve for the WcBiM and Au chips were obtained by finding the maximum absolute value of their derivatives. The steepest slopes for the WcBiM and Au chips were 25.3495%/° at 50.5° and 15.4530%/° at 58.3°, respectively. The WcBiM chip had a slope that was approximately 1.64 times steeper than the Au chip. A comparative analysis of the SPR reflectance curves presented in Figure 3A with the results in Figure 3B indicates that the WcBiM chip was expected to have higher sensitivity than the Au chip in the intensity interrogation detection scheme, as mentioned in the previous section [36]. Thus, the detection of the presence of Aβ42 was carried out by measuring the reflectance using the WcBiM chip at the fixed incident angle (50.5°) where the maximum gradient is located.

### Detection of Aβ42 in the SPR sensor

**Figure 4:**
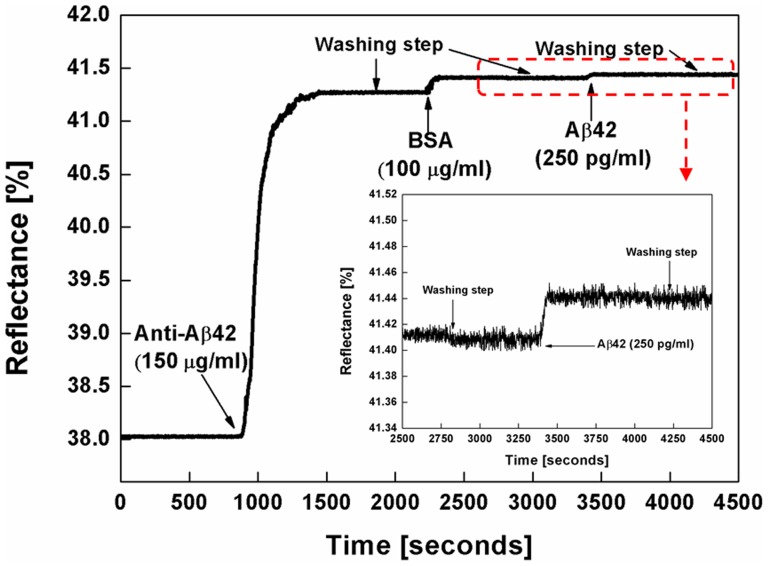
SPR sensorgram. SPR sensorgram of subsequent injection of anti-Aβ42, BSA, and Aβ42 after the SAM formation on the WcBiM chip. Inset: the magnified graph of the sensor response to Aβ42 (250 pg/ml).

**Figure 5:**
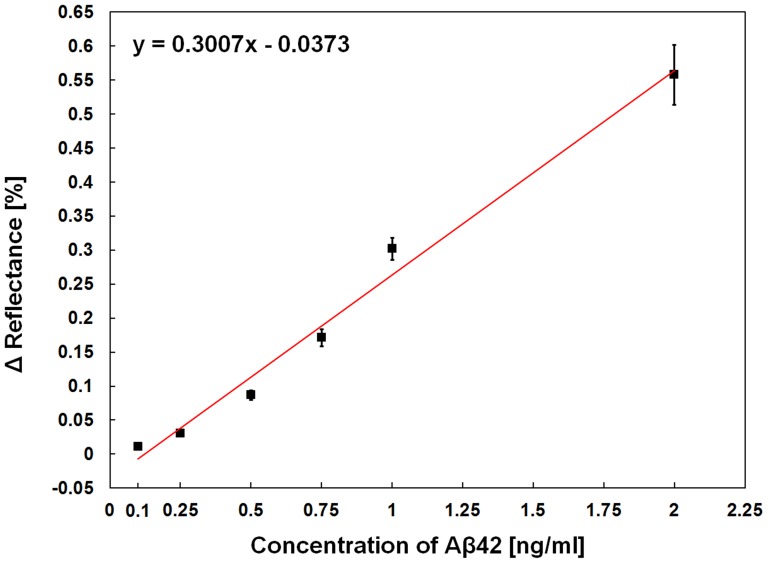
Calibration curve. Calibration curve of the average SPR response to various Aβ42 concentrations ranging from 100 pg/ml to 2,000 pg/ml. The error bars represent the SD of three replicates.

Figure 4 shows the typical sensorgram for the detection of Aβ42. The reflectance at the fixed angle mode is shown as a function of time. In order to selectively detect the presence of Aβ42, anti-Aβ42, BSA, and Aβ42 solutions were sequentially injected into the WcBiM chip after the SAM formation on the WcBiM chip. The average reflectance increments for anti-Aβ42, BSA, and Aβ42 (250 pg/ml) were 3.6182%, 0.1227%, and 0.0305%, respectively, for the three replicates. The magnified graph of the sensor response to Aβ42 (250 pg/ml) is shown in the inset in Figure 4. We utilized the mean value of the stable output signal for 100 seconds as the sensor output signal. For the three replicates, the average response to the Aβ42 in a series of six concentrations (100 pg/ml, 250 pg/ml, 500 pg/ml, 750 pg/ml, 1,000 pg/ml, and 2,000 pg/ml) were 0.0111%, 0.0305%, 0.0867%, 0.1712%, 0.3021%, and 0.5577%, respectively, as shown in Figure 5. The error bars represent the standard deviation (SD) for the three replicates. This concentration range was chosen since 500 pg/ml is the critical concentration needed to diagnose Alzheimer disease, as mentioned earlier. In order for the WcBiM SPR chip to be practical, it is significant to have a linear correlation between the SPR response (changes in the reflectance (ΔR)) and the concentration of Aβ42. From linear regression analysis, the relation of the SPR response with the Aβ42 in the range of 100 pg/ml to 2,000 pg/ml was linear. For this linear calibration curve, the slope of the line was 0.3007 [%/(ng/ml)], and the correlation coefficient of the system was about 99%. The limit of detection (LOD) is defined as the lowest concentration of the analyte which commonly produces the output signal higher than three-times the SD of the reading of the absence of the analyte [40]. The SD of the SPR sensor in the absence of the Aβ42 was 0.00283%. Thus, three-times the SD of the reading of the absence of the Aβ42 was 0.00849%. Therefore, the aforementioned increments for the various Aβ42 concentrations were judged to be a meaningful signal.

### Control experiment

**Figure 6:**
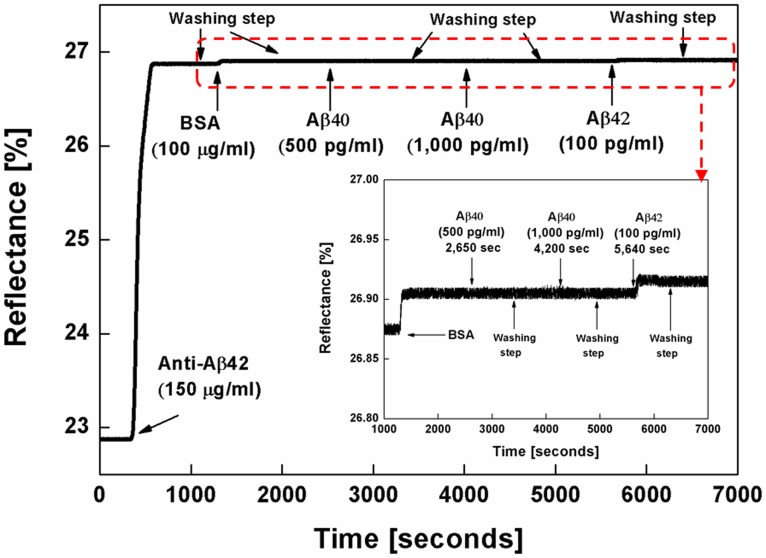
SPR sensorgram for the control experiment.

To demonstrate the specificity of the anti-Aβ42-modified surface of the WcBiM chip, a control experiment was carried out. For this purpose, Aβ40 solutions with concentrations of 500 pg/ml and 1,000 pg/ml were injected onto the anti-Aβ42-modified surface of the WcBiM chip. Sequential injection of the anti-Aβ42, BSA, Aβ40 (500 pg/ml, 1,000 pg/ml), and Aβ42 (100 pg/ml) solutions onto the SAM-modified surface of the WcBiM SPR chip was performed, and the SPR sensorgram for the control experiment was acquired, as shown in Figure 6. In this case, the SPR responses to the anti-Aβ42, BSA, Aβ40 (500 pg/ml, 1,000 pg/ml), and Aβ42 (100 pg/ml) solutions were 3.9989%, 0.0308%, 0.0001%, 0.0002% and 0.0093% respectively. When considering the amount of time required for the sample to flow through the fluidic channel from the inlet to the detection spot, the SPR signals corresponding to Aβ40 solutions with concentrations of 500 pg/ml and 1,000 pg/ml were expected to start at 2,650 and 4,200 seconds. It was observed that there was almost no reflectance change to the Aβ40 since the SPR response to the 1,000 pg/ml of Aβ40 (0.0002%) was less than 0.00849%. Although the Aβ42 solution had a lower concentration than the Aβ40 solution and was injected after the Aβ40 solution, the SPR response to the 100 pg/ml of Aβ42 solution was distinctly increased. It is verified that the anti-Aβ42-modified sensor chip has a high specificity with Aβ42 and does not react with other proteins such as Aβ40.

Compared to the conventional detection methods such as MS and STM, the SPR sensor with the WcBiM configuration offers real-time monitoring and label-free detection that reduces time or cost since it does not require any additional chemical process. Angular interrogation mode and Au SPR chip has been widely adopted in the conventional SPR system. However, it has a limitation for detecting target at trace level concentration. In order to enhance sensitivity or resolution, the easiest way to modify the sensor system is change of the materials. Among the SPR detection schemes, the most effective enhancement in sensitivity or resolution by changing the materials can occur in the intensity interrogation mode. The intensity interrogation mode measures the changed reflectance at fixed incident angle with the steepest slope in the SPR reflectance curve. As shown in Figure S3, in the intensity interrogation mode, the narrower line with of SPR reflectance curve, the larger change in the reflectance. The design of the WcBiM SPR system was experimentally demonstrated, and the line width of the reflectance curve of the WcBiM film was narrower than the line width of the conventional Au film as shown in the Figure 3. From analysis of biomolecular interaction, the sensitivity of the WcBiM SPR chip in the intensity interrogation detection scheme was much higher than that of the single Au SPR chip [36]. Thus, the SPR sensor with the WcBiM chip in a fixed angle mode can detect the Aβ42 around 500 pg/ml of the concentration, which is critical for diagnosing Alzheimer disease.

## Conclusions

In this work, we have investigated the characteristics of the WcBiM chip for the detection of Aβ42 in order to diagnose AD. The line width of the reflectance for the WcBiM chip was narrower than the line width of the conventional Au chip. Thus, the reflectance curve of the WcBiM chip has a steeper gradient, resulting in a significant change in the reflectance to the minute concentration of the analyte. After appropriate surface modification on the WcBiM SPR chip surface with SAM, anti-Aβ42 with high-density was immobilized on the WcBiM SPR chip surface for the detection of a low concentration of Aβ42. The SPR responses, the average changes in the reflectance to the Aβ42 at the concentrations of 100 pg/ml, 250 pg/ml, 500 pg/ml, 750 pg/ml, 1,000 pg/ml, and 2,000 pg/ml for three replicates were 0.0111%, 0.0305%, 0.0867%, 0.1712%, 0.3021%, and 0.5577% respectively. It can be concluded that these values were judged to be a meaningful signal by analyzing the LOD. From linear regression analysis, the calibration curve of the SPR response to Aβ42 with a concentration ranging from of 100 pg/ml to 2,000 pg/ml was linear. From the control experiment, it is proven that the SPR sensor system readily distinguished the Aβ42 from the control analyte (Aβ40) due to a specific receptor and its merit of selectivity. Therefore, the result obtained from the SPR sensor with the WcBiM chip shows that Aβ42 can be successfully detected at the critical concentration (500 pg/ml) needed to diagnose AD. In addition, the combination of the WcBiM SPR chip with the proper surface modification in the intensity interrogation detection scheme is able to provide for the detection of other disease-related biomarkers.

## Supporting Information

Figure S1 **SPR reflectance curve for the WcBiM and bimetallic configurations.** The Ag/Au ratio of both configurations was equal.
(TIF)

Figure S2 **Increment for Anti-Aβ42 ranging from 10 µg/ml to 200 µg/ml.** Output response to various concentrations of the anti-Aβ42 solution for the high-density immobilization measurement. The change of 10 RU corresponds to 0.001° of the SPR angle change.
(TIF)

Figure S3 **Comparison of the reflectance change in two SPR reflectance curves.** A) Narrower FWHM in the SPR reflectance curve. B) Broader FWHM in the SPR reflectance curve.
(TIF)
