# Supplementary figures and images for: Detection of Amyloid-β42 Using a Waveguide-Coupled Bimetallic Surface Plasmon Resonance Sensor Chip in the Intensity Measurement Mode
Source: PLoS One. 2014 Jun 9;9(6):e98992. doi: 10.1371/journal.pone.0098992 (PMC4049661; doi:10.1371/journal.pone.0098992)

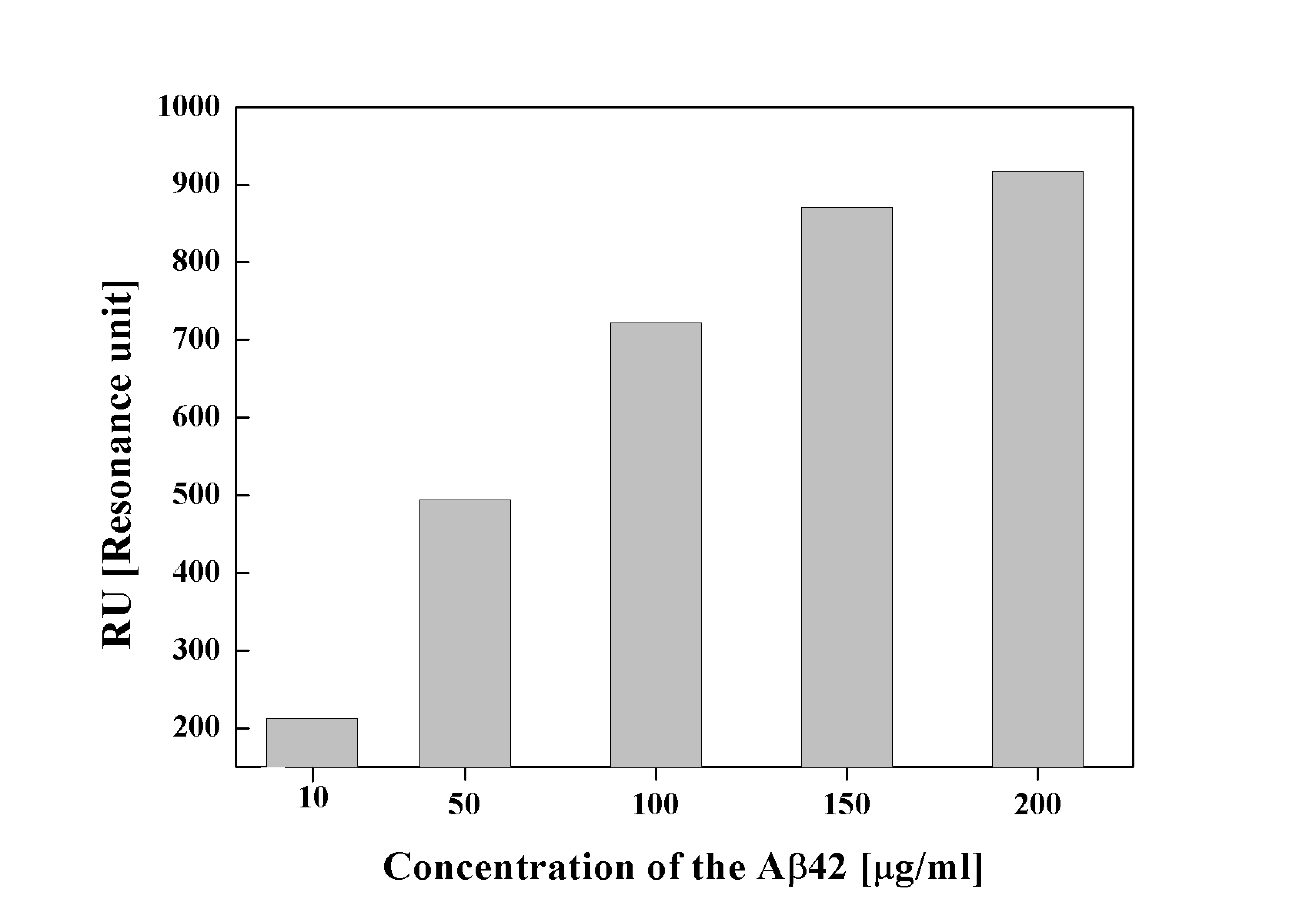

Supplement: Figure S2 — Increment for Anti-Aβ42 ranging from 10 µg/ml to 200 µg/ml. Output response to various concentrations of the anti-Aβ42 solution for the high-density immobilization measurement. The change of 10 RU corresponds to 0.001° of the SPR angle change. (TIF) [file pone.0098992.s002.tif]

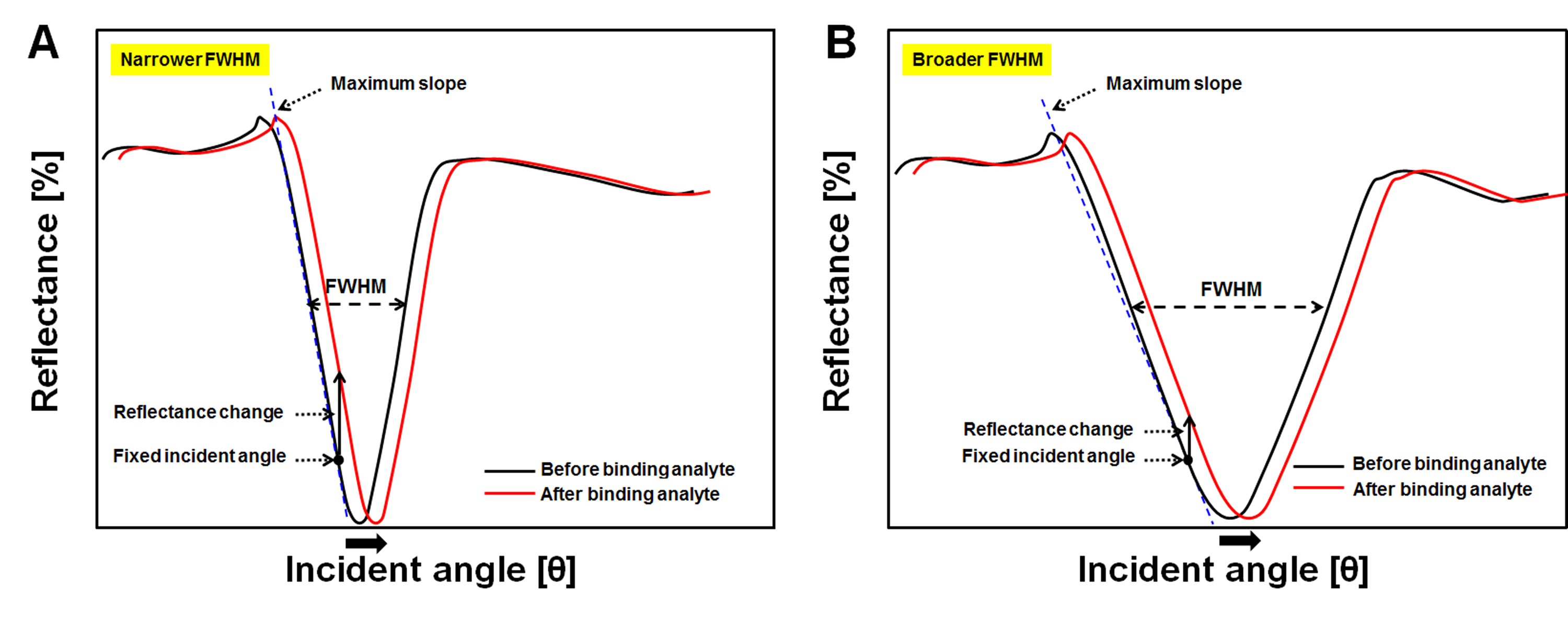

Supplement: Figure S3 — Comparison of the reflectance change in two SPR reflectance curves. A) Narrower FWHM in the SPR reflectance curve. B) Broader FWHM in the SPR reflectance curve. (TIF) [file pone.0098992.s003.tif]
